# Malaria, HIV and Malnutrition among Internally Displaced People in Mozambique During COVID-19 Pandemic: Results from a Community-Based Intervention

**DOI:** 10.5334/aogh.3969

**Published:** 2022-12-05

**Authors:** Francesco Di Gennaro, Edocardo Occa, Lucy Ramirez, Claudia Marotta, Francesco Vladimiro Segala, Jaime Santana, Sergio Cotugno, Roberta Papagni, Giovanna De Meneghi, Emanuela De Vivo, Cati Braque, Giorgia Guelfi, Samo Manhica, Ilaria Di Nunzio, Nelson Foquisso, Giacomo Opocher, Francesca Tognon, Annalisa Saracino, Giovanni Putoto

**Affiliations:** 1 Clinic of Infectious Diseases, University of Bari, University Hospital Policlinico, 70121 Bari, Italy; 2 Operational Research Unit, Doctors with Africa-Cuamm, Padua, Italy; 3 Doctors with Africa CUAMM Mozambique, MZ; 4 University of Bologna, Italy

**Keywords:** Community surveillance, internally displaced people, COVID-19, Malnutrition, Mozambique

## Abstract

**Background:**

The spread of COVID-19 poses an unprecedented challenge to care delivery in post-disaster and conflict situations. In Mozambique, the 2019 cyclone Idai and the violence by Non-State-Armed-Groups devastated the province of Sofala and Cabo Delgado respectively and led to the displacement of thousands of people living in poor and overcrowded conditions. The pandemic has further aggravated the situation. Doctors with Africa CUAMM (University college for aspiring missionary doctors) implemented surveillance activities in these regions between October 2020 and September 2021. The aim of this study is to give an overview of the prevalence of malaria, malnutrition, COVID-19 related symptoms and access to HIV testing.

**Methods:**

Data were collected in targeted internally displaced people (IDP) sites in Sofala and Cabo Delgado province between 31st January and 25th September 2021. The tool used enabled to assess COVID-19 symptoms, risk of HIV infection, malaria cases and malnutrition in children under five.

**Results:**

The project reached 93 503 people. During the study period, 13.6% people reported at least one symptom suggestive of COVID-19 infection. Malaria Rapid Diagnostic Tests (RDT) were administered to 86% of the recruited people (n = ?), with a positive diagnosis in the 4.5% of them (n = ?). Among the recruited Internally Displaced Persons (IDP), 23.1% were considered eligible for HIV screening, but only 1.4% were referred for testing. Acute malnutrition was found in 6.3% of children screened and, among these, a higher prevalence of concurrent COVID-19 symptoms was reported.

**Discussion:**

Our study highlights the importance of mass clinical screening for COVID-19 infection in this target population to enact prevention behavior, although this may not be enough, due to the pivotal role played by asymptomatic transmissions. Considering the overlap of the symptoms of COVID-19 and malaria, a combined diagnostic algorithm is urgently needed to avoid underdiagnosing malaria. Moreover, the high prevalence of respiratory symptoms in malnourished children confirmed the known correlation between malnutrition and respiratory infection. Finally, access to HIV screening needs to be implemented, given the high prevalence of people with HIV risk factors to avoid diagnostic delay.

**Conclusions:**

Population-specific needs make necessary to develop new screening methods that respond to the specific characteristics of the target population.

## Introduction

Internally displaced people (IDP) are among the most vulnerable populations since they struggle to access essential health, water, and sanitation services including clean water [1]. In March 2019, Sofala province was hit by the cyclone Idai, resulting in the displacement of thousands of people [2], while the violence carried out by Non-State Armed Groups in Cabo Delgado province led to the displacement of hundreds of thousands of people. Apart from the insufficient access to proper sanitation and vaccination, internally displaced people (IDP) live in situations of overcrowding, are more vulnerable to HIV infection [3], and routinely face surges in malaria and malnutrition [4,5]. In times of pandemics, HIV, malnutrition and malaria control in vulnerable populations is further threatened by supply-chain interruptions, health resources overload and decision-making adaptation [6].

In this scenario, the advent of severe acute respiratory syndrome coronavirus 2 (SARS-CoV-2), brought significant disruption. In March 2020, the first cases of the COVID-19 pandemic were reported in Mozambique. Pandemics usually have a major impact on vulnerable communities and, although COVID-19 is well-known in Mozambique, myths about avoidance and cures must be dispelled. As happened in other sub-Saharan countries, the infection rate rose in two peaks, occurring from January to April 2021 and July to September 2021 [7]. The spectrum of clinical manifestations of COVID-19 ranges from pauci- or asymptomatic infections to severe respiratory failure and death [8]. The most common clinical symptoms of COVID-19 include loss of smell, loss of taste, cough, runny nose, fatigue, fever, and chest pain and, in some patients, SARS-CoV2 acute infection resolves in a wide spectrum of long-term signs and symptoms known as “long COVID”. Even though symptom-screening alone is considered unreliable in COVID-19 identification and surveillance [9], symptomatic individuals are significantly more likely to test positive [10].

From October 2020 to September 2021, Doctors with Africa CUAMM (University college for aspiring missionary doctors) implemented a community-based surveillance intervention with support from the United Nations Population Fund (UNFPA) in Mozambique. The initiative was funded by Norway as part of a project that aimed to contribute to Mozambique’s efforts in preparing and responding to the COVID-19 pandemic, with a focus on mitigating its consequences on the lives of women and girls affected by cyclone Idai and Kenneth in Sofala and armed conflicts in Cabo Delgado. CUAMM’s interventions targeted internally displaced people (IDPs) accommodated in 12 resettlement sites established since 2019. In those areas, the project piloted the community surveillance strategy developed by the Mozambican Ministry of Health. The aim of this study is to report test-positivity rate and prevalence of malaria, malnutrition, COVID-19-related symptoms and access to HIV testing among people living in resettlement camps. Data will be used as baseline assessment for future intervention.

## Methods

### Study setting

In Mozambique, the IDP site is usually composed of a limited number of areas/neighborhoods (bairros), each one accommodating people coming from the same community or neighboring villages, in order to guarantee linguistic and cultural closeness, kinship, cohesion and uniformity.

For the intervention, CUAMM engaged with teams of 15 community-based volunteers, or “activists”, in each district (totaling 90 in the 2 provinces). Activists were purposely selected for being part of the target communities and for their proficiency in local languages and willingness to be trained in surveillance, data collection processes, and administration of screening tools for malaria, HIV and malnutrition.

Symptom screening was carried out based on the indications contained in the 2020 “Community Intervention Strategy for the fight against the COVID-19 pandemic” [11] defined by the Ministry of Health and in which CUAMM actively contributed to its design and drafting.

In accordance with local authorities, each activist was assigned to a specific geographical area, thus avoiding overlapping data and duplication. Here, activists screened and interviewed random samples of the population, mapped each household and cross-checked the information with the census data provided by local authorities. Data was then shared with the supervisor on a weekly basis and entered into an electronic Excel database.

The intervention was implemented in IDP communities located in Sofala and Cabo Delgado provinces, Mozambique. In Sofala, 12 resettlement sites distributed in 3 districts were targeted: Nhamatanda (IDP sites of Ndeja and Segredo), Dondo (Mutua and Setembro) and Beira (Macurungo and Munhava). In Cabo Delgado, study intervention took place in the districts of Montepuez (IDPs sites of Mapupulo and Nicuapa), Chiúre (Meculane and Marrupa) and Ancuabe (Nanjua A and Nanjua B).

## Data collection and analysis

Data were collected in targeted IDP sites between 31^st^ January to 25^th^ September 2021, using data collection tools developed by the Ministry of Health. The first sites where outreach activities were implemented were Nhamatanda and Sofala province, where the data collection lasted for up to 34 weeks (see Table 1). The tool enabled the assessment of COVID-19 symptoms, HIV testing, malaria cases and screening for malnutrition in children under 5 years of age. In addition to screening, the intervention included community support in developing hand washing devices from readily available materials. Furthermore, study staff conducted individual and group awareness sessions on the prevention of COVID-19, HIV and malaria.

**Table 1:** Data collection details and demographic characteristics of people who accessed the service and diseases screening in Sofala and Cabo Delgado.


		SOFALA				CABO DELGADO				TOTAL OVERALL
	
		NHAMATANDA	DONDO	BEIRA	TOTAL SOFALA PROVINCE	MONTEPUEZ	CHIÚRE	ANCUABE	TOTAL CABO DELGADO PROVINCE	

Estimated population in IDP sites		14 126	16 522	23 883	54 531	14 502	13 918	12 654	41 074	**95 605**

Data collection starting date		January 31^st^, 2021	February 7^th^, 2021	February 21^st^, 2021		February 28^th^, 2021	March 14^th^, 2021	March 14^th^, 2021		

Total weeks of data collection		34 weeks (from Epi Week 5 to 38)	33 weeks (from Epi Week 6 to 38)	31 weeks (from Epi Week 8 to 38)		30 weeks (from Epi Week 6 to 38)	28 weeks (from Epi Week 11 to 38)	28 weeks (from Epi Week 11 to 38)		

Total population reached		13 259	16 418	23 882	53 559	14 416	13 068	12 460	39 944	**93 503**

Sex	Male	6 097 (46.0)	7 366 (44.9)	11 080 (46.4)	24 543 (45.8)	6 654 (46.2)	6 168 (47.2)	5 939 (47.7)	18 761 (47.0)	**43 304 (46.3)**

	Female	7 162 (54.0)	9 052 (55.1)	12 802 (53.6)	29 016 (54.2)	7 762 (53.8)	6 900 (52.8)	6 521 (52.3)	21 183 (53.0)	**50 199 (53.7)**

Age	<5	1 986 (15.0)	2 668 (16.3)	2 610 (10.9)	7 264 (13.6)	2 515 (17.4)	2 160 (16.5)	1 606 (12.9)	6 281 (15.7)	**13 545 (14.5)**

	5-24	6 140 (46.3)	8 392 (51.1)	11 441 (47.9)	25 973 (48.5)	6 032 (41.8)	5 074 (38.8)	5 349 (42.9)	16 455 (41.2)	**42 428 (45.4)**

	25-44	3 101 (23.4)	3 623 (22.1)	7 337 (30.7)	14 061 (26.3)	3 345 (23.2)	3 730 (28.5)	3 516 (28.2)	10 591 (26.5)	**24 652 (26.4)**

	>44	2 032 (15.3)	1 735 (10.6)	2 494 (10.4)	6 261 (11.7)	2 524 (17.5)	2 104 (16.1)	1 989 (16.0)	6 617 (16.6)	**12 878 (13.8)**

Covid-19 symptoms	Cough	437 (3.3)	1 031 (6.3)	230 (1.0)	1 698 (3.2)	3 418 (23.7)	3 262 (25.0)	2 389 (19.2)	9 069 (22.7)	**10 767 (11.5)**

	Fever	640 (4.8)	983 (6.0)	142 (0.6)	1 765 (3.3)	3 491 (24.2)	2 792 (21.4)	2 524 (20.3)	8 807 (22.0)	**10 572 (11.3)**

	Running nose	140 (1.1)	406 (2.5)	63 (0.3)	609 (1.1)	1 494 (10.4)	2 340 (17.9)	167 (1.3)	4 001 (10.0)	**4 610 (4.9)**

	Difficulty breathing	96 (0.7)	373 (2.3)	20 (0.1)	489 (0.9)	1 249 (8.7)	2 014 (15.4)	369 (3.0)	3 632 (9.1)	**4 121 (4.4)**

	At least one symptom	882 (6.7)	1 137 (6.9)	355 (1.5)	2 374 (4.4)	3 107 (21.6)	4 272 (32.7)	2 927 (23.5)	10 306 (25.8)	**12 680 (13.6)**

Malaria	Total tested	11 783 (88.9)	15 822 (96.4)	21 993 (92.1)	49 598 (92.6)	9 728 (67.5)	9 320 (71.3)	11 747 (94.3)	30 795 (77.1)	**80 393 (86.0)**

	Positive	416 (3.1)	48 (0.3)	157 (0.7)	621 (1.2)	999 (6.9)	2 071 (15.8)	514 (4.1)	3 584 (9.0)	**4 205 (4.5)**

HIV	Screened	11 475 (86.5)	7 071 (43.1)	23 882 (100)	42 428 (79.2)	13 908 (96.5)	12 264 (93.8)	12 464 (100)	38 636 (96.7)	**81 064 (86.7)**

	Eligible for testing	1 563 (11.8)	31 (0.2)	18 763 (78.6)	20 357 (38.0)	71 (0.5)	1 139 (8.7)	0 (0.0)	1 210 (3.0)	**21 567 (23.1)**

	Referred for test	30 (0.2)	10 (0.1)	222 (0.9)	262 (0.5)	81 (0.6)	908 (6.9)	65 (0.5)	1 054 (2.6)	**1 316 (1.4)**

	Self-revealed HIV	76 (0.6)	791 (4.8)	465 (1.9)	1 332 (2.5)	277 (1.9)	974 (7.5)	212 (1.7)	1 463 (3.7)	**2 795 (3.0)**

	Self-revealed HIV on ART	66 (0.5)	624 (3.8)	465 (1.9)	1 155 (2.2)	252 (1.7)	918 (7.0)	207 (1.7)	1 377 (3.4)	**2 532 (2.7)**

Malnutrition*	Screened	1 344 (67.7)	2 251 (84.4)	2389 (91.5)	5 984 (82.4)	2 148 (85.4)	1 926 (89.2)	1 424 (88.7)	5 498 (87.5)	**11 482 (84.8)**

	Malnourished	31 (1.6)	100 (3.7)	33 (1.3)	164 (2.3)	109 (4.3)	198 (10.3)	386 (24.0)	693 (11.0)	**857 (6.3)**

	Malnourished with COVID symptoms	27 (1.4)	44 (1 6)	4 (0.2)	75 (1.0)	44 (1.7)	210 (0.1)	345 (21.5)	599 (9.5)	**674 (5.0)**


* As denominator only <5 population reached has been considered.

Recorded COVID-19 symptoms included fever, cough, runny nose and difficulty in breathing. In case of clinical suspicion, malaria rapid diagnostic tests (RDT) were immediately administered. HIV and TB patients were monitored and sensitized on therapy adherence, but follow-up data were not included in this study. The nutritional status of children aged 6 to 59 months was assessed using MUAC (Mid Upper Arm Circumference).

COVID-19 suspected cases were referred to the nearest health facility for further testing and treatment. Once validated by the health facility, data collected in this study were used for the drafting of monthly reports that were shared by the District Health Department and then transferred to the National Health Information System for Monitoring and Evaluation (SISMA), managed by the Ministry of Health. Reports were also shared with District and Provincial Health Departments and UNFPA.

## Results

The project reached 93 503 people, including 53 559 (29 016 females and 24 543 males) people living in Sofala and 39 944 (21 183 females and 18 761 males) in Cabo Delgado province. The modal age group was 5–14 years. In total, women represented 53.7% of the population (Table 1).

During the study period, a total of 12 680 (13.6%) people reported at least 1 symptom suggestive of COVID-19 infection, respectively 2 374 (4.4%) and 10 306 (25.8%) living in Sofala and Cabo Delgado. Overall, data shows a higher prevalence of COVID-19 symptoms in Cabo Delgado, peaking in March-April and August-September 2021, especially in Chiure district. The most frequently observed symptoms were cough and fever, both present in 11% (n = 10 767) of the recruited people.

**Figure 1:**
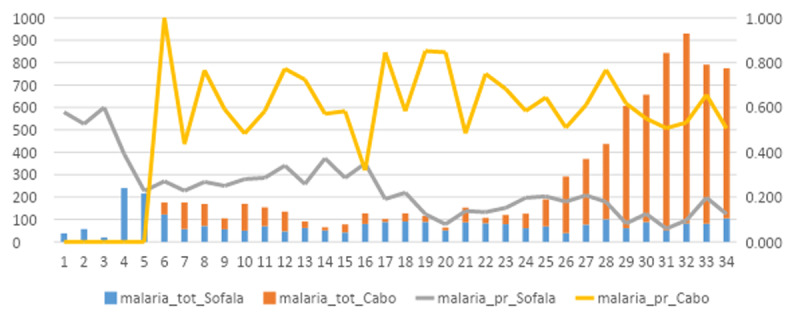
**Total number of malaria cases and test positivity rate.** Vertical bars show the total number of malaria cases diagnosed among internally-displaced people in Sofala (blue) and Cabo Delgado (orange) provinces. Continuous lines show the test-positivity rate for malaria in Sofala (grey) and Cabo Delgado (yellow) provinces during COVID-19 pandemic.

Concerning malaria, a total of 621 out of 2 664 (23.3%) of subjects living in Sofala and 3 584 out of 6 160 (58.2%) in Cabo Delgado tested positive during the study period. Figure 1 shows the temporal trend of malaria testing and incidence in the two provinces.

Among people living in Sofala, 38.0% of recruited subjects (n = 20 357) were considered eligible for an HIV screening, but only 262 people (0.5%) were referred for testing, while in Cabo Delgado, 1 210 (3.0%) were eligible and 1 054 (2.6%) were referred for testing.

**Figure 2:**
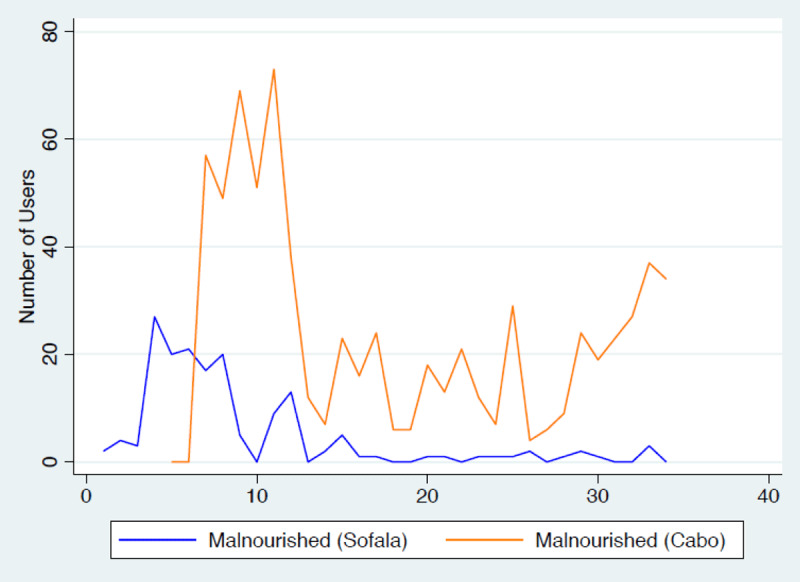
**Total number of malnutrition cases.** Total number of internally displaced children <5 diagnosed with malnutrition in Sofala (blue line) and Cabo Delgado (orange) provinces during the COVID-19 pandemic. X axis: Epi week; Y axis: number of children diagnosed with malnutrition per week.

Eighty-four percent of the 13 545 children under the age of 5 that were reached by community outreach were screened for malnutrition and, in the Sofala region, 164 (2.7%) were diagnosed with acute malnutrition. Interestingly, in Sofala, 75% of the children with acute malnutrition reported at least 1 concurrent sign or symptom of COVID-19 (45.7%). In the Cabo Delgado region, acute malnutrition was diagnosed in 693 out of 5 498 (12.6%) children under5, with a prevalence of concurrent COVID-19 symptoms as high as 86.4% (n = 599) of the children presenting at least 1sign and symptom of COVID-19. In this study, the malnutrition rate was higher in Cabo Delgado than in Sofala, with the highest incidence between the 5th and 15th Epi week (Figure 2).

## Discussion

This study follows the lead of community mobilization to promote health from a wider perspective. During COVID-19 times, among internally displaced people, active community engagement promotes disease awareness and mitigates the lack of healthcare workforce, thereby contrasting the diversion of health expenditure from primary healthcare to COVID-19 treatment [12]. In this context, internally displaced people are particularly vulnerable to COVID-related healthcare disruption, since several factors – such as poor living conditions, societal isolation, and instability of healthcare supply – converge in affecting the delivery of sustained disease control interventions [13]. In terms of infectious diseases, since the beginning of the pandemic, African countries have witnessed a large increase in the disability-adjusted life years (DALY) related to HIV, TB, and malaria, combined with a generalized decrease in case reporting.

Since the beginning of the pandemic, although the high prevalence of hard-to-reach populations, cultural barriers, and diagnostic tool unavailability has been recognized as critical challenges for COVID-19 management in Africa [14,15,16], the recent experience with Ebola fostered a rapid, diffuse community-based response [17]. In this context, our study highlights the importance of mass clinical screening, as we detected 13% of our population had at least 1 COVID-19 symptom. Although we were not able to report disease incidence, the wavelike trend of people positive for COVID-19 clinical screening may suggest that a prompt adoption of prevention behavior (such as isolation) based exclusively on clinical suspicion could help to reduce infection spread. Nevertheless, since asymptomatic transmission plays a pivotal role in COVID-19 spread [8], relying solely on clinical symptoms may be misleading.

In low resource settings, case management is further complicated by the overlapping of COVID-19 and malaria clinical presentation, as fever, joint pain, fatigue, and headache are well-recognized symptoms of both diseases [9,18]. A combined diagnostic algorithm allowing healthcare workers to consider malaria and COVID-19 coinfection is urgently needed [19], as well as studies addressing the combined impact of both diseases among vulnerable populations [20]. In our study, we administered malaria RDT to 86% of the recruited people, which led to a positive diagnosis in 4.5% of the total population and, interestingly, about one-third of the people who reported at least one COVID-19 symptom tested positive for malaria. Although the effect of malaria on COVID-related outcomes is still a matter of debate [21,22], these findings stress the importance of combining mass screening for COVID-19 in malaria service delivery in endemic areas [23].

On the other hand, it is widely confirmed that malnutrition and pneumonia are strictly related, as respiratory infections are one of the major complications of undernutrition, and wasting and stunting increase the mortality from pneumonia by about 15-fold [24]. On its own, pneumonia represents 14% of the overall under 5 mortality [25]. In this context, we understand that the COVID-19 pandemic sheds new light on pneumonia epidemiology and therefore on how we need to approach pneumonia as a disease. For this reason, our study included screening for malnutrition, reaching 84.8% of children under 5 years old in this population.

While evidence is growing that, worldwide, children are less likely to develop severe forms of COVID-19 [26], in African countries a substantially lower rate of disease is observed. Several epidemiological factors (such as the protective effect of other endemic infections or vaccinations) are being studied. Nevertheless, since the beginning of the pandemic, malnutrition proved to be bound to COVID-19 disease both for being a risk factor for severe disease and for being a consequence of the COVID-induced social crisis [27,28]. In our population, among the 6.5% of under 5 children diagnosed with acute malnutrition, about 80% presented at least one symptom of COVID-19. However, as stated before, we were not able to assess the proportion of those symptoms attributable to SARS-CoV2 and, given the increased risk of pneumonia experienced by malnourished children [24], they may be the manifestation of other respiratory infections due to, for instance, respiratory syncytial virus (RSV), human metapneumovirus, *S. pneumoniae* or *S. aureus*.

Finally, our project included referrals for HIV screening. Access to HIV testing represents a constant challenge, as mistrust and fear often hampered screening initiatives. In our study, we notice that the community mobilization contributed to promoting access to HIV testing, as 86.7% of people were reached and counselled by study interviewers. On the basis of the interview, one-fourth of the enrolled subjects were eligible for the test, as they fit the risk criteria identified by our tool. Nevertheless, just 6% of our population got tested for HIV. This low rate stresses the existence of barriers to accessing HIV testing among IDPs, which, in turn, may be a consequence of the long distance to the first HIV testing facility, which should be prioritized when planning future interventions. In our project, the only possible referral to HIV testing service was the mobile service managed by our team, which visited the camps. In this context, Omam LA et al [29]. describe a similar experience with mobile-clinic among IDPs in Cameroon, and the authors conclude that this approach represents a promising tool to overcome difficulties due to the COVID-related constraints in access to health services.

Nevertheless, a common finding in HIV projects among IDPs is that the absence of a structural service for HIV screening represents a major challenge for test delivery to all eligible people [30]. Thus, a reaction strategy to SARS-CoV2 outbreak, along with the implementation of COVID-19 mitigation initiatives, should entail the adaptation of existing services for delivering care for all the major causes of death and morbidity, including malnutrition and HIV-malaria coinfections [31,32].

We recognize some limitations in our study. For example, the lack of testing capacity for COVID-19, no consideration can be made to the actual verified case number of COVID-19 resulting from the clinical screening alone.

HIV testing depended on a mobile team without-reach activity. This makes it difficult to understand the real percentage of people who would have actually benefited from the combined screening and would have gone to be tested for HIV.

## Conclusion

The COVID-19 pandemic in the African context can develop different scenarios in respect of what has been reported in other continents. Population-specific needs make it necessary to develop new screening methods that respond to the specific characteristics of the target population. Thus, non-healthcare personnel can be trained for a first symptom-based screening, but, considering the importance of asymptomatic transmission of SARS-CoV-2, this needs to be associated with proper microbiologic testing.

The use of funds and social resources mobilized for COVID-19 is an important chance to improve healthcare to fight against HIV and other transmissible diseases. In regions where malaria is endemic, the disease should be ruled out in all cases of febrile illness. For this reason, combined point-of-care testing for both COVID-19 and malaria, along with HIV sensitization and screening for malnutrition, play a pivotal role in community-based interventions like the one described here. In low-resource settings, the diversion of financial and human resources to initiatives exclusively focusing on COVID-19 care may, instead of bringing solutions, decrease the overall health status of vulnerable populations such as IDP.
